# Nematodes and the effect of seasonality in grassland habitats of South Africa

**DOI:** 10.21307/jofnem-2020-118

**Published:** 2020-12-14

**Authors:** Chantelle Girgan, Gerhard du Preez, Mariette Marais, Antoinette Swart, Hendrika Fourie

**Affiliations:** 1 Nematology Unit, Biosystematics, Agricultural Research Council-Plant Health and Protection (ARC-PHP), Private Bag X134, Queenswood, 0121, South Africa; 2 Unit for Environmental Sciences and Management, North-West University (NWU), Private Bag X6001 Potchefstroom, 2520, South Africa; 3 Agricultural Research Council – Tropical and Subtropical Crops (ARC-TSC), Private Bag X11208, Nelspruit, 1200, South Africa; 4 Department of Zoology, University of Johannesburg, P.O. Box 524, Auckland Park, Johannesburg, 2006, South Africa

**Keywords:** Ecosystem functioning, Food web status, Grassland habitats, Seasonal variation, Soil ecology

## Abstract

Nematodes in South Africa have mainly been studied for their diversity and agricultural importance. However, the ecological status of nematodes and the effect of seasonal variation in local grasslands remain unknown. For this reason, a nematode study was conducted in the Telperion Nature Reserve and represented the first ecological study in a natural grassland area in South Africa. In total, 104 soil samples were collected during four consecutive seasons from 2015 until 2016 in three habitats, viz. (i) open grassland, (ii) shrubland with rocky outcrops, and (iii) riparian zone. From these the nematode community structure and soil ecosystem status were studied. In total, 93 genera from 50 families were recorded with herbivores and bacterivores being the most abundant trophic groups in all three habitats. Linear mixed models revealed that season had an overwhelmingly dominant impact on the condition, food web status, and functioning of the soil ecosystems with pairwise comparisons indicating that significantly higher values were recorded during winter. Interestingly, this seasonal shift can largely be attributed to fluctuations in the populations of only a few nematode groups (namely *Aporcelaimellus*, Dorylaimidae, *Iotonchus*, and *Mononchus*) with high colonizer-persister values. Although the reason for the higher abundance of specific nematode groups recorded during the winter is not explicitly clear, it is possibly linked to reduced competition from other soil fauna. This study clearly shows that further investigations are required to better understand the dynamics of grassland ecosystems.

Nematodes occupy most terrestrial habitats on earth (Liu et al., 2019), even the furthest reaches of caves (Du Preez et al., 2017) and deep underground mines (Borgonie et al., 2011). Estimates also indicate that nematodes represent 80% of all multicellular organisms (Eisenhauer and Guerra, 2019; Van Den Hoogen et al., 2019). However, despite their omnipresent distribution and dominating abundance, many nematode communities are poorly studied with the majority of species remaining undescribed (Archidona-Yuste et al., 2016). This is especially true for many habitats in South Africa since most nematode-related studies are focused on agricultural systems and nematodes of economic importance (i.e. crop pests) (Procter, 2012; Fourie et al., 2017).

Grasslands, for example, host diverse soil ecosystems, represent more than 40% of Earth’s terrestrial surface area and provide essential ecosystem services including the provision of food, storage of carbon, mitigating droughts and floods, and erosion control (Hewins et al., 2018; Bengtsson et al., 2019; Gibson and Newman, 2019). However, although the structure and ecological status of grassland nematode communities have been investigated in some parts of the world (Ekschmitt et al., 2001; Biederman and Boutton, 2009; Kergunteuil et al., 2016), a literature survey revealed no ecological reports on the nematode communities of South African grasslands.

Studying the nematode communities of grasslands is not only relevant from an ecological perspective, but also from a conservation perspective. Nematodes are valuable indicators of soil ecosystem disturbance affected by, for example, agricultural activities and climate change (Ferris and Bongers, 2009; Zhong et al., 2017; Zhang et al., 2020). Therefore, studying the nematode communities of natural, undisturbed grasslands will generate valuable baseline data for monitoring disturbance and implementing conservation policies. This would be especially valuable for South Africa as the country’s grasslands are under continuous threat from overgrazing, cultivation, and urban expansion (Haddad and Butler, 2018). Woody (or bush) encroachment, driven by rising temperatures and increasing atmospheric carbon dioxide levels, also presents a major threat (Skowno et al., 2017). This while only 2.0 to 2.8% of South Africa’s grasslands are protected within conserved areas (Carbutt et al., 2011; Haddad and Butler, 2018).

When studying terrestrial ecosystems, it is also important to consider seasonal variation as water availability, temperature, and vegetative growth can have an important effect on the structure and functioning of soil ecosystems (Bongers and Ferris, 1999; Cardoso et al., 2016; Song et al., 2016; Siebert et al., 2020). Song et al. (2016), for example, showed that higher precipitation rates significantly increased nematode abundance and richness in a grassland ecosystem. Bakonyi et al. (2007), in turn, found that the effects of soil drying and warming on nematode communities were dependant on the presence of vegetation with the smallest effect recorded in grasses. Ultimately, understanding how seasonal variation affects soil ecosystems is necessary in order to accurately assess and monitor the potential threats posed by anthropogenic activities and climate change.

For these reasons, this study was undertaken and aimed at (i) studying the nematode community structure in the open grassland, shrubland with rocky outcrops and riparian zone habitats of the Telperion Nature Reserve and (ii) investigating the effect of seasonal-induced changes on the condition, food web status and functioning of the soil ecosystems.

## Materials and methods

### Site description

**Figure 1:**
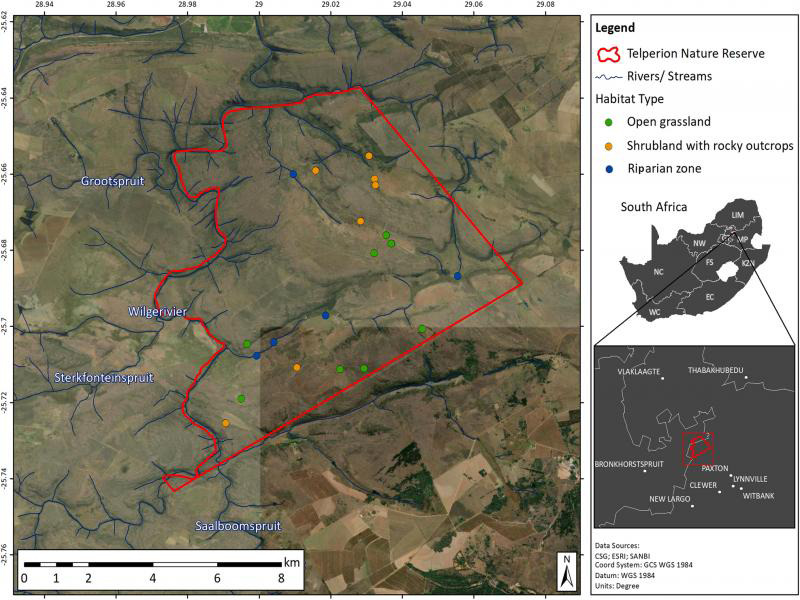
Satellite image of Telperion Nature Reserve (South Africa) indicating the sampling locations associated with the open grassland, shrubland with rocky outcrops and riparian zone habitats.

The study was conducted in the Telperion Nature Reserve (Mpumalanga province, South Africa) (Fig. 1) that has a total surface area of 9,061.3 ha. This reserve is located in the Rand Highveld Grassland (of which only 1% is conserved) and is dominated by grassy plains interspersed with rocky outcrops and woody plant species (Grobler, 1999). The greater region is characterized by strong summer rainfall (570-730 mm per annum) and dry and cold winters (frost may occur frequently). This is a fire prone ecosystem with high lightning flash densities making lightning-induced fires common. The lithology of Telperion Nature Reserve is dominated by Arenite-Conglomerate, producing dystrophic or mesotrophic soils with some red soils, as well as rocky areas with miscellaneous soils (Grobler, 1999). Three streams flow through the reserve that originates from higher lying wetlands and sponge areas.

### Rainfall and temperature data

Climatic data (rainfall and temperature) from January 2014 to December 2016 were obtained from the South African Weather Service (www.weathersa.co.za). The total amount of rain for 90 days prior to each sampling event (as listed in the following section) was calculated, while the mean, minimum, and maximum daily temperatures were extracted for the same period. Since sampling was based on climatic factors (as listed in the following section) and thus not evenly dated over 1 year, this 90-day period was considered before each sampling event.

### Site selection and sample collections

Within Telperion Nature Reserve three main terrestrial habitats, namely, (i) open grassland (OG), (ii) shrubland with rocky outcrops (SRO), and (iii) riparian zone (RZ) were identified for investigation. Sites within each habitat were selected based on accessibility as some areas were restricted and/or not accessible by vehicle or foot. The elevation of the sites in the each habitat ranged as follows: OG: 1333 to 1482 m, SRO: 1361 to 1480 m, and RZ: 1304 to 1383 m above sea level. Ultimately, 26 sampling sites (Fig. 1) were selected and included: 11 OG, 10 SRO sites, and 5 RZ sites.

In total, 104 soil samples (one per site per season) were collected from the three terrestrial habitats during four consecutive seasons [Winter (11 June 2015), Spring (30 November 2015), Summer (24 February 2016), and Autumn (19 April 2016)]. These sampling dates were selected based on climatic patterns through consultation with the reserve manager. Winter samples were the first to be collected when frost was at its peak. Spring sampling followed the first rains, which due to a drought in South Africa came later than normally expected. Summer sampling was undertaken when grasses reached maturity and produced seeds, while Autumn samples were collected before the first frost occurred.

Multiple discrete soil samples (500 cm^3^ each) for the analysis of nematode community structure were collected from each site up to a depth of 20 cm using a garden trowel, which was cleaned between sampling sites to prevent contamination. Samples were collected beneath plants (if present). For the analysis of selected soil properties (soil texture and total organic carbon), a sub-sample was collected from each site. The latter was performed only during the summer (February 2016) sampling interval as the selected soil properties were unlikely to change significantly during the sampling period. All the collected samples were labeled and transported in cool boxes to the Nematology Unit of the Agricultural Research Council – Plant Health and Protection (Roodeplaat, South Africa). Samples were stored at 10°C until further analysis.

### Nematode extraction, preservation, and identification

Nematodes were extracted from 250 cm^3^ soil samples using an adapted decanting and sieving method, followed by a sugar flotation method (Marais et al., 2017). The nematodes were then fixed in a heated 4% formaldehyde and 1% propionic acid (FPG) solution (Netscher and Seinhorst, 1969), dehydrated in a glycerin solution (Seinhorst, 1962), and mounted in glycerin on permanent glass slides using a wax ring (Marais et al., 2017). Nematodes were counted using a De Grisse counting dish (De Grisse, 1963) and identified to genus level using an Olympus BX53F microscope. Taxonomic classification was based on Maggenti et al. (1988) and Geraert (2019) for Tylenchina; Kleynhans et al. (1996) for Heteroderidae; Hunt (1993) for Aphelenchida; Decraemer (1995) and Duarte et al. (2010) for Trichodoridae; Andrássy (2009) for Dorylaimidae; and Andrássy (2005, 2007) for other free-living nematodes. Feeding groups were assigned, based on Yeates et al. (1993), as herbivores (He), bacterivores (Ba), fungivores (Fu), omnivores (Om), or predators (Pr). Nematodes were also assigned a cp-value based on Bongers and Bongers (1998).

### Physical and biological properties of soil samples

The selected physical and biological properties were analyzed by Eco-Analytica (North-West University, South Africa) as follows: total organic carbon (C) content of the soil samples were determined using the loss-on-ignition method (Donkin, 1991) and the soil texture as described by Laker and Dupreez (1982).

### Statistical analysis

Rarefaction curves were used to compare taxa richness between habitats. This was achieved by calculating the sample-based Mao Tau estimator (with inter- and extrapolation) using Estimate S 9.1 Software Package. Furthermore, differences in the abundance of nematode trophic groups between habitats were investigated by pooling samples from across seasons. Abundance values were log_10_(*x* + 1) transformed and visualized, while statistical significance between trophic groups and habitats was inferred using two-way analysis of variance (ANOVA) and Tukey’s multiple comparisons tests. These analyses were performed using Graphpad Prism 6 Software Package.

Selected nematode-based indices (i.e. maturity index 2-5, enrichment index, structure index, channel index, basal index, and metabolic footprints) were used to quantify the condition, food web status, and functioning of soil ecosystems (Ferris et al., 2001; Ferris and Bongers, 2009; Ferris, 2010). These indices were calculated using the Nematode Indicator Joint Analyses (NINJA) online tool (Sieriebriennikov et al., 2014). The Graphpad Prism 6 Software Package was used to create a violin plot of the maturity index 2-5, a measure of soil ecosystem condition. Also, values of the structure and enrichment indices were used to plot a faunal analysis and characterize the food web status of the studied soil ecosystems (Ferris et al., 2001). Furthermore, linear mixed models with pairwise comparisons were used to determine if the independent variables, i.e. season (Winter, Spring, Summer, and Autumn) and habitat (OG, SRO, and RZ) significantly affected (singularly and interactively) the calculated nematode-based indices. Prior to this analysis the nematode-based indices were log transformed (where needed). All linear mixed models were performed using SPSS Statistics 25 Software Package.

Lastly, a redundancy analysis (RDA) was used to investigate the relationship between the response (nematode-based indices) and explanatory variables [(factors (seasons and habitats) and numeric variables (soil texture and total organic carbon)]. Predictor effects of the listed explanatory variables were calculated using a Monte-Carlo permutation test (Šmilauer and Lepš, 2014). These multivariate analyses were performed and illustrated on a biplot using Canoco 5 Software Package. Significance for all univariate and multivariate analyses was regarded at *p* < 0.05.

## Results

### Environmental conditions

**Figure 2:**
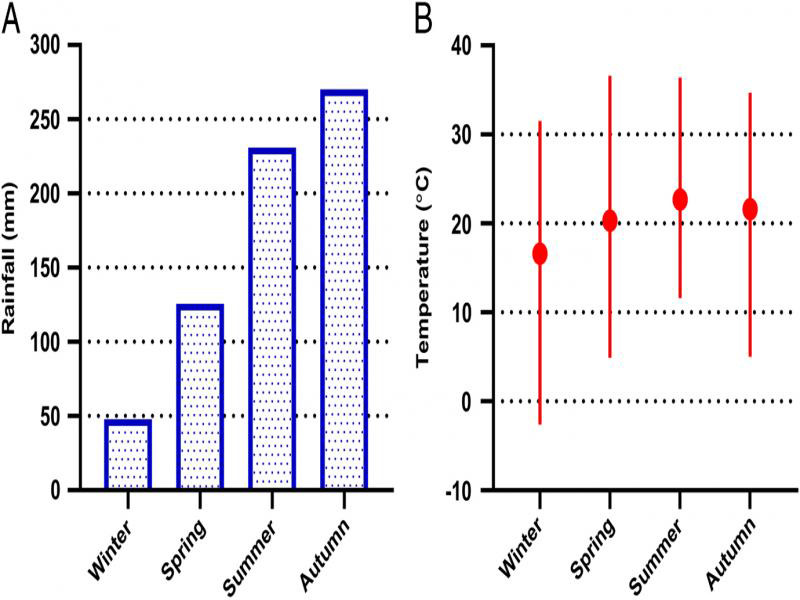
The (A) total rainfall and (B) temperature (mean, minimum, and maximum) for the 90 days prior to each sampling event at Telperion Nature Reserve (South Africa).

The total rainfall for 90 days prior to each sampling event is illustrated in Figure 2A. This shows a general increase over time with the lowest and highest rainfall recorded prior to the Winter and Autumn sampling events, respectively. However, it is important to note that South Africa experienced a drought in 2015. According to the South African Weather Service (www.weathersa.co.za), the Telperion Nature Reserve region received only 388 mm from January to December 2015. By contrast, the recorded annual rainfall in 2014 and 2016 were 794 and 748 mm, respectively.

Temperature (mean, minimum, and maximum) values are illustrated in Figure 2B with the lowest and highest mean temperatures recorded, as expected, prior to the Winter and Summer sampling events, respectively. The largest range between minimum (−2.6°C) and maximum (31.5°C) values were recorded before Winter sampling. Finally, the soil texture and organic carbon content of the collected and analyzed soil samples are provided as Supplementary material (Table S1).

**Table S1.** Investigated soil properties measured at the open grassland, shrubland with rocky outcrops and riparian zone habitats in the Telperion Nature Reserve (Mpumalanga province, South Africa).

Habitat	Sand (%)	Silt (%)	Clay (%)	Organic carbon (%)
Open grassland	91.77 ± 3.03	3.75 ± 2.33	4.47 ± 0.94	0.78 ± 0.24
Shrubland with rocky outcrops	86.45 ± 4.44	6.99 ± 3.13	6.54 ± 2.08	1.65 ± 0.84
Riparian zone	86.48 ± 7.18	8.18 ± 5.16	5.33 ± 2.24	1.59 ± 1.26

**Note:** Mean ± standard deviation values are reported.

### Nematode community structure

Nematode communities associated with the studied habitats were investigated in terms of their taxonomic composition and richness, as well as their trophic abundance. In total, 93 genera, representing 50 families, were recorded in samples collected from Telperion Nature Reserve (Supplementary material Tables S2–S6) and were representative of the five major nematode trophic groups, namely, herbivores (Table S2), bacterivores (Table S3), fungivores (Table S4), omnivores (Table S5), and predators (Table S6). The largest number of taxa were found in the shrubland with rocky outcrops (SRO) habitat (77 genera), followed by the riparian zone (RZ) (74 genera) and open grasslands (OG) (68 genera) habitats. In terms of the number of recorded genera per trophic group, bacterivores dominated with 30 genera recorded in both the SRO and RZ habitats, while 24 bacterivore genera were found in the OG habitat. This was followed by herbivores (OG: 23; SRO: 23; RZ: 20), predators (OG: 11; SRO: 14; RZ: 13), fungivores (OG: 8; SRO: 9; RZ: 8), and omnivores (OG: 2; SRO: 2; RZ: 3). The results also showed that some nematode genera were present in all three habitats during all four seasons. This included *Helicotylenchus, Rotylenchulus, Scutellonema, Tylenchus, Xiphinema* (herbivores, Table S2); *Acrobeles, Acrobeloides, Panagrolaimus*, *Prismatolaimus, Zeldia* (bacterivores; Table S3); *Aphelenchoides, Diphtherophora* (fungivores; Table S4), and Dorylaimidae (omnivores, Table S5).

**Table S2.** Occurrence and abundance of herbivores in 250 cm^3^ soil samples obtained from open grassland, shrubland with rocky outcrops and riparian zone sites in the Telperion Nature Reserve (Mpumalanga province, South Africa) as part of an ecological study that was conducted over four seasons from 2015 to 2016.

		Open grassland				Shrubland with rocky outcrops				Riparian zone			
Family	Genus or Family	Winter	Spring	Summer	Autumn	Winter	Spring	Summer	Autumn	Winter	Spring	Summer	Autumn
Anguinidae (He1)	*Subanguina*	1.36 ± 4.52	0	2.73 ± 9.05	0.45 ± 1.51	0	0	0	0	0	1.00 ± 2.24	0	1.00 ± 2.24
Belondiridae (He5)	*Axonchium*	0.91 ± 3.02	3.64 ± 4.52	0	0.91 ± 3.02	0	5.00 ± 12.69	3.00 ± 7.89	0.50 ± 1.58	0	0	1.00 ± 2.24	0
Belonolaimidae (He3)	*Tylenchorhynchus*	1.82 ± 6.03	0	0	30.91 ± 65.91	18.00 ± 56.92	0.50 ± 1.58	25.00 ± 36.82	8.00 ± 13.98	0	0	0	0
Criconematidae (He3)	Criconematidae^a^	1.82 ± 4.62	5.00 ± 13.42	0.91 ± 3.02	0.91 ± 3.02	3.00 ± 3.50	3.00 ± 6.32	1.00 ± 3.16	4.50 ± 9.56	1.00 ± 2.24	0	1.00 ± 2.24	0
	*Criconema*	0.91 ± 3.02	0	0	0	0	0	0	0	0	0	0	2.00 ± 4.47
	*Criconemoides*	6.36 ± 15.67	10.45 ± 17.24	7.27 ± 11.91	8.18 ± 22.39	0.50 ± 1.58	9.00 ± 21.19	5.00 ± 14.14	0	5.00 ± 11.18	5.00 ± 11.18	1.00 ± 2.24	0
	*Hemicriconemoides*	0	1.82 ± 6.03	0	0	0	1.00 ± 3.16	0	0	0	1.00 ± 2.24	0	0
	*Hemicycliophora*	5.91 ± 19.60	2.27 ± 7.54	0.45 ± 1.51	1.82 ± 6.03	1.50 ± 4.74	0.50 ± 1.58	0	1.00 ± 3.16	165.00 ± 301.58	13.00 ± 29.07	69.00 ± 154.29	1.00 ± 2.24
Dorylaimellidae (He5)	*Dorylaimellus*	58.18 ± 98.82	6.36 ± 13.80	8.64 ± 18.04	11.36 ± 31.23	21.50 ± 25.28	12.50 ± 20.85	8.50 ± 16.51	12.00 ± 25.19	0	5.00 ± 11.18	1.00 ± 2.24	55.00 ± 122.98
Heteroderidae (He3)	*Meloidogyne*	4.09 ± 10.68	2.27 ± 7.54	1.82 ± 4.05	25.45 ± 71.40	154.50 ± 350.55	13.00 ± 19.32	11.50 ± 16.67	8.00 ± 17.03	0	162.00 ± 342.90	45.00 ± 81.70	54.00 ± 120.98
Hoplolaimidae (He3)	Hoplolaimidae^a^	6.36 ± 10.51	7.27 ± 16.94	0	2.27 ± 4.10	15.50 ± 26.29	14.00 ± 26.01	10.50 ± 31.49	0	2.00 ± 4.47	0	1.00 ± 2.24	0
	*Helicotylenchus*	38.64 ± 43.25	139.09 ± 246.41	42.27 ± 112.37	40.45 ± 64.67	52.00 ± 82.27	67.50 ± 102.29	31.50 ± 57.40	61.50 ± 12.83	6.00 ± 13.42	83.00 ± 185.59	3.00 ± 6.71	73.00 ± 139.45
	*Rotylenchulus*	34.55 ± 71.57	50.91 ± 128.78	31.82 ± 47.29	55.91 ± 125.89	34.50 ± 92.87	46.00 ± 143.72	53.00 ± 139.92	96.00 ± 275.77	2.00 ± 4.47	28.00 ± 59.85	3.00 ± 6.71	1.00 ± 2.24
	*Rotylenchus*	29.55 ± 42.51	21.36 ± 38.61	0	16.36 ± 22.59	18.50 ± 35.04	5.50 ± 11.65	0.50 ± 1.58	7.00 ± 10.59	15.00 ± 28.28	0	0	8.00 ± 7.89
	*Scutellonema*	110.45 ± 131.25	172.73 ± 231.38	38.64 ± 71.91	35.45 ± 36.77	44.00 ± 51.47	64.00 ± 92.04	131.00 ± 343.24	21.00 ± 23.78	19.00 ± 26.55	40.00 ± 58.74	35.00 ± 57.77	137.00 ± 275.83
Leptonchidae (He4)	*Xiphinemella*	6.82 ± 22.61	0	1.36 ± 3.23	5.45 ± 9.34	4.50 ± 14.23	2.50 ± 7.91	0.50 ± 1.58	5.50 ± 12.57	0	0	1.00 ± 2.24	2.00 ± 4.47
Longidoridae (He5)	*Longidorus*	0	0	0	0	0	0	0	0	0	3.00 ± 6.71	0	0
	*Xiphinema*	6.82 ± 13.65	2.27 ± 4.10	7.27 ± 10.81	5.91 ± 10.68	20.22 ± 31.09	3.00 ± 5.37	11.50 ± 16.17	11.00 ± 16.96	3.00 ± 4.47	2.00 ± 2.74	6.00 ± 13.42	1.00 ± 2.24
Pratylenchidae (He3)	*Pratylenchus*	10.45 ± 19.42	22.73 ± 44.74	2.27 ± 3.44	5.91 ± 13.38	27.00 ± 34.50	16.00 ± 35.81	15.50 ± 29.67	5.50 ± 10.66	0	0	0	0
Psilenchidae (He2)	*Psilenchus*	0.91 ± 2.02	0	0	0	0	0	3.00 ± 9.49	0	31.00 ± 69.32	7.00 ± 15.65	15.00 ± 33.54	0
Trichodoridae (He4)	*Nanidorus*	0	2.73 ± 9.05	0	0	0	2.50 ± 5.40	1.50 ± 4.74	0.50 ± 1.58	5.00 ± 7.07	18.00 ± 28.42	313.00 ± 683.17	28.00 ± 49.32
	*Paratrichodorus*	0	0	0	0	2.00 ± 6.32	0	0	0	0	6.00 ± 13.42	0	0
	Trichodoridae^a^	0	0.45 ± 1.51	0	0	0	0	0	0	0	0	0	0
	*Trichodorus*	0	0	0	0	0	0.50 ± 1.58	0	0	0	0	0	0
Tylenchidae (He2)	*Filenchus*	0	0	0	0	0	0	0	0	0	41.00 ± 80.96	0	0
	*Coslenchus*	0	2.73 ± 9.05	0.45 ± 1.51	0	0	0.50 ± 1.58	0	0	0	0	0	0
	Tylenchidae^a^	0.45 ± 1.51	1.36 ± 3.23	0	0	0	2.50 ± 7.91	0	0.50 ± 1.58	1.00 ± 2.24	16.00 ± 30.50	0	0
	*Tylenchus*	77.73 ± 73.33	33.64 ± 40.38	15.91 ± 36.53	21.82 ± 24.21	121.50 ± 65.75	18.00 ± 18.59	14.00 ± 11.01	26.00 ± 27.37	139.00 ± 144.80	85.00 ± 100.93	246.00 ± 222.64	189.00 ± 263.71
Tylencholaimidae (He4)	*Chitwoodius*	0	0	0	0.45 ± 1.51	0.50 ± 1.58	0	0	0	0	0	0	0
Tylenchulidae (He3)	*Meloidoderita*	3.64 ± 12.06	0	0	0	0	0	0	0	0	0	0	0
	*Tylenchulus*	0	0	0.45 ± 1.51	0	0	2.00 ± 6.32	0	0	0	0	0	0
Total herbivores		407.73 ± 194.43	489.09 ± 476.05	162.27 ± 214.97	270.00 ± 206.95	539.00 ± 383.70	289.00 ± 240.71	326.50 ± 363.48	268.50 ± 289.86	394.00 ± 353.77	516.00 ± 496.61	741.00 ± 922.38	552.00 ± 734.72

**Notes:** Mean ± standard deviation values are reported. ^a^Genus could not be identified.

**Table S3.** Occurrence and abundance of bacterivores in 250 cm^3^ soil samples obtained from open grassland, shrubland with rocky outcrops and riparian zone sites in the Telperion Nature Reserve (Mpumalanga province, South Africa) as part of an ecological study that was conducted over four seasons from 2015 to 2016.

		Open grassland				Shrubland with rocky outcrops				Riparian zone			
Family (functional guild)	Genus or Family	Winter	Spring	Summer	Autumn	Winter	Spring	Summer	Autumn	Winter	Spring	Summer	Autumn
Alaimidae (Ba4)	*Alaimus*	98.64 ± 91.33	17.73 ± 19.15	2.27 ± 11.07	12.73 ± 19.02	92.00 ± 57.07	21.50 ± 22.74	18.50 ± 22.61	57.50 ± 63.56	10.00 ± 14.14	22.00 ± 49.19	1.00 ± 2.24	0
	*Amphidelus*	0	0	0	0	0	0.51 ± 1.58	5.50 ± 7.62	5.00 ± 11.55	0	0	0	1.00 ± 2.24
Aphanolaimidae (Ba3)	*Aphanolaimus*	0	0	0	0	0	0	0	0	1.00 ± 2.24	0	0	0
Cephalobidae (Ba2)	Cephalobidae^a^	0	13.18 ± 18.07	87.73 ± 192.33	113.64 ± 198.92	0	19.00 ± 45.26	32.00 ± 38.82	36.50 ± 45.03	0	0	3.00 ± 2.74	10.00 ± 14.14
	*Acrobeles*	122.27 ± 112.48	69.09 ± 103.46	36.82 ± 55.51	160.45 ± 192.57	51.50 ± 69.76	52.50 ± 123.52	18.00 ± 39.24	24.50 ± 36.40	3.00 ± 6.71	47.00 ± 64.58	32.00 ± 54.15	13.00 ± 16.43
	*Acrobeloides*	60.45 ± 41.44	20.45 ± 21.62	25.00 ± 53.06	23.64 ± 23.67	24.00 ± 43.83	66.50 ± 152.19	43.00 ± 103.98	21.50 ± 39.86	25.00 ± 30.00	52.00 ± 52.51	62.00 ± 107.62	63.00 ± 105.69
	*Cephalobus*	3.18 ± 9.02	42.27 ± 59.22	54.09 ± 47.21	97.73 ± 171.37	18.00 ± 50.01	64.50 ± 118.75	116.00 ± 272.01	63.50 ± 76.52	0	75.00 ± 148.66	58.00 ± 57.07	26.00 ± 28.59
	*Cervidellus*	0	8.64 ± 15.51	7.27 ± 15.55	7.73 ± 10.09	0	8.00 ± 15.67	3.50 ± 5.30	4.50 ± 6.43	0	50.00 ± 111.80	1.00 ± 2.24	5.00 ± 8.66
	*Chiloplacus*	20.45 ± 22.52	4.09 ± 6.25	3.18 ± 10.55	0.91 ± 3.02	13.00 ± 15.49	13.50 ± 17.49	2.00 ± 6.32	16.00 ± 44.02	60.00 ± 128.65	4.00 ± 8.94	1.00 ± 2.24	0
	*Eucephalobus*	0	0.91 ± 3.02	1.82 ± 4.62	0	0	121.00 ± 380.88	8.50 ± 16.34	5.50 ± 11.17	0	9.00 ± 20.12	167.00 ± 365.10	40.00 ± 61.64
	*Heterocephalo-bellus*	0	0	0	0.45 ± 1.51	0	0	0	0.50 ± 1.58	0	0	0	0
	*Seleborca*	53.64 ± 90.11	57.73 ± 84.42	89.55 ± 186.83	57.73 ± 71.39	8.00 ± 22.01	18.50 ± 46.19	7.50 ± 6.35	14.50 ± 16.24	0	17.00 ± 30.33	6.00 ± 10.84	1.00 ± 2.24
	*Zeldia*	28.18 ± 32.96	35.45 ± 77.05	2.73 ± 5.18	13.18 ± 16.17	6.00 ± 7.38	8.00 ± 12.74	8.50 ± 13.75	4.00 ± 6.58	3.00 ± 6.71	22.00 ± 49.19	2.00 ± 4.47	4.00 ± 8.94
Chromadoridae (Ba3)	*Punctodora*	0	0	0	0	0	1.00 ± 3.16	0	0	9.00 ± 12.45	0	0	0
Chronogasteridae (Ba3)	*Chronogaster*	0	0	0	0.45 ± 1.51	0.50 ± 1.58	0	2.00 ± 4.83	0.50 ± 1.58	279.00 ± 509.20	12.00 ± 14.40	95.00 ± 212.43	28.00 ± 57.18
Cyatholaimidae (Ba4)	*Achromadora*	0	0	4.55 ± 15.08	0	0	0	0	0	9.00 ± 15.17	2.00 ± 2.74	18.00 ± 40.25	0
Diplogasteridae (Ba1)	*Butlerius*	0	0	0	0	1.50 ± 3.37	4.50 ± 14.23	0	0	3.00 ± 6.71	2.00 ± 4.47	0	0
Diplogasteroididae (Ba1)	Diplogasteroididae^a^	0	0	0	0	0	0	0	0	0	0	14.00 ± 31.30	1.00 ± 2.24
Elaphonematidae (Ba3)	*Elaphonema*	8.64 ± 17.76	1.82 ± 4.62	8.64 ± 15.51	12.73 ± 42.21	6.50 ± 11.56	0	7.00 ± 22.14	1.50 ± 3.37	0	0	0	0
Mesorhabditidae (Ba1)	*Mesorhabditis*	0	3.18 ± 10.55	0	8.18 ± 27.14	0	12.50 ± 32.68	1.50 ± 3.37	2.00 ± 6.32	0	14.00 ± 21.91	2.00 ± 4.47	0
Metateratocepha-lidae (Ba3)	*Euteratocephalus*	0	0	0	0	0	0	0	0	5.00 ± 8.66	0	0	0
Monhysteridae (Ba2)	*Monhystera*	1.82 ± 6.03	0	0	0	2.50 ± 4.86	2.00 ± 6.32	0	0	8.00 ± 13.04	4.00 ± 6.52	3.00 ± 4.47	6.00 ± 10.84
	*Monhystrella*	0	0	0	0	00		0	0	0	0	0	2.00 ± 4.47
Neodiplogastridae (Ba1)	*Fictor*	0	0	0	0	0	0	0	0.50 ± 1.58	2.00 ± 4.47	0	0	0
	*Koerneria*	0	0	0	0	2.00 ± 6.32	0	0	0	0	1.00 ± 2.24	3.00 ± 6.71	0
Odontolaimidae (Ba3)	*Odontolaimus*	0	0	0	0	0	0	0	0	2.00 ± 4.47	0	0	0
Osstellidae (Ba2)	*Drilocephalobus*	0	0	18.18 ± 50.71	37.73 ± 71.46	0	0	3.50 ± 4.74	12.00 ± 14.57	0	0	0	1.00 ± 2.24
Panagrolaimidae (Ba1)	*Panagrolaimus*	26.36 ± 25.31	15.00 ± 23.02	0.91 ± 3.02	17.27 ± 35.31	17.50 ± 14.19	17.50 ± 39.32	12.50 ± 23.83	4.00 ± 7.38	39.00 ± 39.12	15.00 ± 21.21	19.00 ± 21.33	6.00 ± 6.52
Plectidae (Ba2)	*Anaplectus*	0	0	0	0	0	0	0	0.50 ± 1.58	0	0	0	0
	*Ceratoplectus*	0	0	0	0.91 ± 2.02	10.50 ± 33.20	4.50 ± 11.17	2.50 ± 7.91	11.00 ± 25.03	0	2.00 ± 4.47	0	0
	*Plectus*	5.45 ± 12.34	0	0	0	4.00 ± 12.65	0	1.50 ± 2.42	3.50 ± 11.07	4.00 ± 4.18	1.00 ± 2.24	2.00 ± 4.47	1.00 ± 2.24
	*Wilsonema*	3.18 ± 7.83	0.45 ± 1.51	0	1.36 ± 2.34	6.50 ± 10.55	1.50 ± 2.42	2.50 ± 5.40	6.50 ± 17.17	0	6.00 ± 13.42	1.00 ± 2.24	1.00 ± 2.24
Prismatolaimidae (Ba3)	*Prismatolaimus*	0.91 ± 2.02	0.91 ± 2.02	0.91 ± 3.02	3.18 ± 10.55	6.50 ± 11.80	7.00 ± 11.35	7.50 ± 13.99	7.00 ± 12.52	3.00 ± 2.74	5.00 ± 11.18	6.00 ± 13.42	3.00 ± 4.47
Rhabditidae (Ba1)	*Cruznema*	0	0	0	0	0	0	0	0.50 ± 1.58	0	0	0	0
	Rhabditidae^a^	0.45 ± 1.51	0	0	0	0	0	0	0	0	0	0	0
	*Rhabditis*	1.36 ± 3.23	2.27 ± 4.67	0	3.18 ± 5.13	18.00 ± 31.55	12.50 ± 34.42	2.00 ± 4.22	5.00 ± 11.55	7.00 ± 13.04	6.00 ± 8.94	0	0
Rhabdolaimidae (Ba3)	*Rhabdolaimus*	0	0	0.45 ± 1.51	0	0	0	0.50 ± 1.58	0	0	0	0	0
Teratocephalidae (Ba3)	*Teratocephalus*	0.45 ± 1.51	0.45 ± 1.51	0	0	0	4.00 ± 11.01	0	0	4.00 ± 6.52	0	0	0
Total bacterivores		435.45 ± 275.30	293.64 ± 291.33	349.09 ± 507.99	573.18 ± 517.51	288.50 ± 147.82	460.50 ± 719.66	306.00 ± 435.38	308.00 ± 126.34	476.00 ± 506.13	368.00 ± 364.02	496.00 ± 603.15	212.00 ± 129.79

**Notes:** Mean ± standard deviation values are reported. ^a^Genus could not be identified.

**Table S4.** Occurrence and abundance of fungivores in 250 cm^3^ soil samples obtained from open grassland, shrubland with rocky outcrops and riparian zone sites in the Telperion Nature Reserve (Mpumalanga province, South Africa) as part of an ecological study that was conducted over four seasons from 2015 to 2016.

		Open grassland				Shrubland with rocky outcrops				Riparian zone			
Family	Genus or Family	Winter	Spring	Summer	Autumn	Winter	Spring	Summer	Autumn	Winter	Spring	Summer	Autumn
Anguinidae (Fu2)	*Ditylenchus*	8.64 ± 13.98	29.55 ± 40.28	28.18 ± 33.86	10.91 ± 13.75	1.50 ± 4.74	20.50 ± 38.18	19.00 ± 19.97	23.50 ± 30.56	0	25.00 ± 34.64	130.00 ± 144.27	110.00 ± 186.38
Aphelenchidae (Fu2)	*Aphelenchus*	15.91 ± 16.71	133.18 ± 190.56	86.36 ± 246.80	24.55 ± 35.67	12.50 ± 13.59	51.50 ± 63.20	80.00 ± 161.57	9.00 ± 9.66	0	19.00 ± 26.08	72.00 ± 102.02	68.00 ± 141.01
Aphelenchoididae (Fu2)	*Aphelenchoides*	8.64 ± 15.34	10.45 ± 15.72	21.36 ± 61.08	5.91 ± 7.01	13.00 ± 23.12	284.50 ± 887.40	16.00 ± 30.26	4.00 ± 6.58	15.00 ± 23.98	13.00 ± 13.04	21.00 ± 20.74	12.00 ± 21.39
Diphtherophoridae (Fu3)	*Diphtherophora*	23.18 ± 22.28	8.64 ± 13.62	5.00 ± 7.42	0.91 ± 2.02	24.00 ± 19.55	3.00 ± 6.32	23.50 ± 67.37	32.50 ± 86.35	9.00 ± 15.17	6.00 ± 13.42	34.00 ± 73.26	32.00 ± 35.81
Leptonchidae (Fu4)	*Leptonchus*	0	1.36 ± 3.23	0.91 ± 3.02	0.91 ± 3.02	0	1.00 ± 2.11	0	0.50 ± 1.58	0	1.00 ± 2.24	0	0
	*Proleptonchus*	0	0	3.18 ± 10.55	0	0	0	0	0.50 ± 1.58	0	0	0	0
Neotylenchidae (Fu2)	Neotylenchidae^a^	5.91 ± 10.20	3.64 ± 5.05	8.64 ± 15.02	10.00 ± 13.04	5.00 ± 15.81	11.50 ± 20.15	12.50 ± 18.45	18.50 ± 19.87	0	17.00 ± 28.20	23.00 ± 51.43	10.00 ± 17.32
Paraphelenchidae (Fu2)	*Paraphelenchus*	0	0	0	0	2.00 ± 6.32	0	0	0	1.00 ± 2.24	0	9.00 ± 20.12	0
Tylencholaimellidae (Fu4)	*Tylencholaimellus*	6.36 ± 11.85	0	0.45 ± 1.51	0.45 ± 1.15	3.50 ± 8.18	0.50 ± 1.58	2.00 ± 6.32	0	0	0	0	1.00 ± 2.24
Tylencholaimidae (Fu4)	Tylencholaimidae^a^	2.73 ± 9.05	0	0	0	0	0	0	0	1.00 ± 2.24	0	0	0
	*Tylencholaimus*	7.27 ± 24.12	1.82 ± 4.05	24.09 ± 65.83	1.36 ± 4.52	7.00 ± 14.76	0	9.00 ± 28.46	0	2.00 ± 4.47	0	8.00 ± 15.25	0
Total fungivores		78.64 ± 54.13	188.64 ± 229.54	178.18 ± 342.99	55.00 ± 41.23	68.50 ± 35.83	372.50 ± 863.64	162.00 ± 179.20	88.50 ± 120.21	28.00 ± 22.53	81.00 ± 93.10	297.00 ± 341.31	233.00 ± 360.60

**Notes:** Mean ± standard deviation values are reported. aGenus could not be identified.

**Table S5.** Occurrence and abundance of omnivores in 250 cm^3^ soil samples obtained from open grassland, shrubland with rocky outcrops and riparian zone sites in the Telperion Nature Reserve (Mpumalanga province, South Africa) as part of an ecological study that was conducted over four seasons from 2015 to 2016.

		Open grassland				Shrubland with rocky outcrops				Riparian zone			
Family	Genus or Family	Winter	Spring	Summer	Autumn	Winter	Spring	Summer	Autumn	Winter	Spring	Summer	Autumn
Dorylaimidae (Om4)	Dorylaimidae^a^	55.91 ± 22.89	20.45 ± 23.07	11.36 ± 19.25	17.27 ± 21.14	49.00 ± 29.23	14.00 ± 14.10	17.50 ± 29.65	16.00 ± 16.96	15.00 ± 12.75	15.00 ± 14.14	6.00 ± 6.52	18.00 ± 40.25
	*Dorylaimus*	0.45 ± 1.51	0.45 ± 1.51	0	0	0.50 ± 1.58	0	1.00 ± 3.16	0	3.00 ± 6.71	0	0	0
	*Mesodorylaimus*	0	0	0	0	3.00 ± 9.49	1.50 ± 4.74	0	2.50 ± 7.91	1.00 ± 2.24	3.00 ± 4.47	1.00 ± 2.24	2.00 ± 4.47
Tylencholaimidae (Om4)	*Discomyctus*	0	0	0	0	0	0	0	0	0	0	0	6.00 ± 13.42
Mydonomidae (Om4)	*Dorylaimoides*	0.91 ± 3.02	0	0	0	0	0	0	0	0	0	0	0
Total omnivores		57.27 ± 23.60	20.91 ± 23.00	11.36 ± 19.25	17.27 ± 21.14	52.50 ± 31.38	15.50 ± 14.990	18.50 ± 29.160	18.50 ± 20.010	19.00 ± 9.62	18.00 ± 16.81	7.00 ± 5.70	26.00 ± 52.73

**Notes:** Mean ± standard deviation values are reported. ^a^Genus not identified.

**Table S6.** Occurrence and abundance of predators in 250 cm^3^ soil samples obtained from open grassland, shrubland with rocky outcrops and riparian zone sites in the Telperion Nature Reserve (Mpumalanga province, South Africa) as part of an ecological study that was conducted over four seasons from 2015 to 2016.

		Open grassland				Shrubland with rocky outcrops				Riparian zone			
Family	Genus or Family	Winter	Spring	Summer	Autumn	Winter	Spring	Summer	Autumn	Winter	Spring	Summer	Autumn
Actinolaimidae (Pr5)	*Neoactinolaimus*	0	0	0	0	0	0	0	0	0	0	2.00 ± 4.47	0
Anatonchidae (Pr4)	*Iotonchus*	4.09 ± 7.01	0.45 ± 1.51	0.45 ± 1.51	0	9.50 ± 12.79	1.50 ± 2.42	3.00 ± 4.22	1.00 ± 3.16	2.00 ± 4.47	0	14.00 ± 31.30	4.00 ± 8.94
Aphelenchoididae (Pr2)	*Seinura*	0	0	0	0	0	0	0	0	0	0	0	44.00 ± 98.39
Aporcelaimidae (Pr5)	Aporcelaimidae^a^	1.36 ± 4.52	0	0	1.82 ± 4.62	0	1.00 ± 3.16	0	0.50 ± 1.58	0	0	0	3.00 ± 6.71
	*Aporcelaimellus*	16.36 ± 16.75	0.91 ± 3.02	5.91 ± 9.95	1.36 ± 4.52	3.50 ± 11.07	3.50 ± 4.12	9.50 ± 13.83	4.50 ± 9.56	2.00 ± 4.47	0	0	0
	*Aporcelaimus*	1.36 ± 3.23	5.00 ± 9.49	5.00 ± 9.22	4.09 ± 12.00	0	3.50 ± 5.80	3.50 ± 11.07	0	1.00 ± 2.24	2.00 ± 2.74	6.00 ± 13.42	19.00 ± 39.75
	*Makatinus*	0	0.91 ± 3.02	0	0	0	0	1.50 ± 4.74	0.50 ± 1.58	0	0	0	0
Ironidae (Pr4)	*Ironus*	0	0	0	0	0	0	0	0	4.00 ± 8.94	0	0	0
Mononchidae (Pr4)	Mononchidae^a^	0.45 ± 1.51	0.45 ± 1.51	0	0	0	0	1.00 ± 3.16	0	0	2.00 ± 2.74	0	0
	*Cobbonchus*	0	0	0	0	0	0	0	0	3.00 ± 4.47	0	0	0
	*Granonchulus*	0	0	0	0	0	1.50 ± 4.74	0	0	0	0	0	0
	*Mononchus*	0.45 ± 1.51	0.45 ± 1.51	0	1.82 ± 6.03	14.00 ± 29.04	0.50 ± 1.58	0	0	2.00 ± 2.74	0	0	0
Mylonchulidae (Pr4)	*Mylonchulus*	0	0	0.91 ± 3.02	0	2.00 ± 4.83	0	0	0	0	0	0	0
Paraxonchiidae (Pr5)	*Paraxhonchium*	4.55 ± 10.60	1.36 ± 3.23	3.64 ± 12.06	0	1.50 ± 4.74	0	3.00 ± 7.89	6.00 ± 14.49	0	0	0	0
Qudsianematidae (Pr4)	Discolaiminae^a^	1.36 ± 4.52	0	0	0	2.00 ± 4.83	0	0	4.00 ± 8.76	0	0	0	0
	*Discolaimoides*	9.09 ± 14.80	2.73 ± 5.18	12.73 ± 25.43	20.91 ± 27.28	1.50 ± 3.37	0	1.00 ± 3.16	9.00 ± 12.87	2.00 ± 2.74	0	0	3.00 ± 6.71
	*Discolaimus*	0.91 ± 3.02	0.45 ± 1.51	0	1.36 ± 4.52	0.50 ± 1.58	2.50 ± 5.40	0.50 ± 1.58	0.50 ± 1.58	0	1.00 ± 2.24	1.00 ± 2.24	2.00 ± 2.74
	*Eudorylaimus*	0	0	0	0	0	0.50 ± 1.58	0	0	0	0	2.00 ± 4.47	0
	*Labronema*	0	1.82 ± 6.03	0	0	0.50 ± 1.58	0.50 ± 1.58	1.00 ± 3.16	0.50 ± 1.58	0	0	6.00 ± 8.94	0
Tobrilidae (Pr4)	*Tobrilus*	0	0	0	0	1.00 ± 3.16	0	0	0	2.00 ± 2.74	1.00 ± 2.24	1.00 ± 2.24	1.00 ± 2.24
Tripylidae (Pr3)	*Tripyla*	0	0	0	0.45 ± 1.51	1.50 ± 4.74	0.50 ± 1.58	1.00 ± 3.16	0	0	0	0	0
Total predators		40.00 ± 28.81	14.55 ± 20.30	28.64 ± 24.40	31.82 ± 27.04	37.5 ± 34.56	15.5 ± 11.20	24.5 ± 21.67	26.5 ± 24.23	18.00 ± 13.51	6.00 ± 4.18	32.00 ± 39.15	76.00 ± 131.79

**Notes:** Mean ± standard deviation values are reported. ^a^Genus could not be identified.

**Figure 3:**
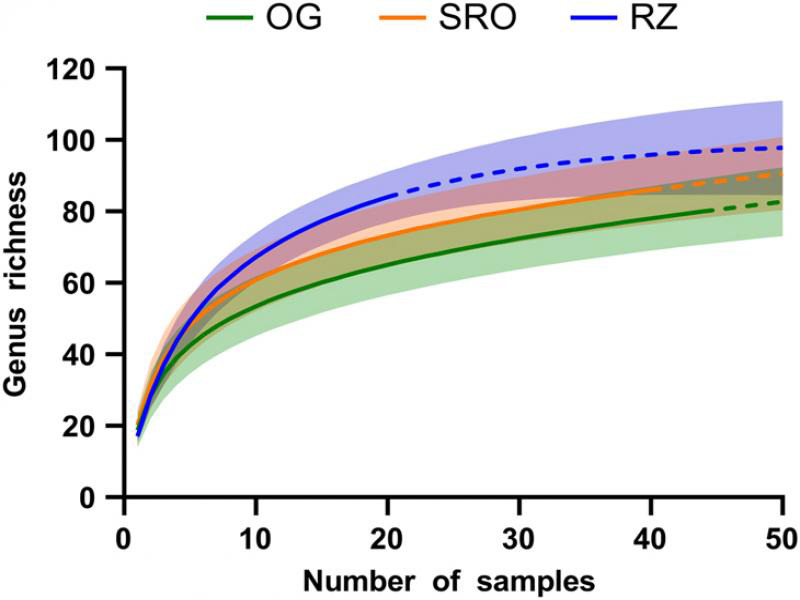
Rarefaction curves illustrate the sample-based observed (solid line) and extrapolated (dotted line) Mau Tau richness of nematode genera at the open grassland (OG), shrubland with rocky outcrops (SRO), and riparian zone (RZ) habitats in Telperion Nature Reserve (South Africa). The shaded bands represent the 95% confidence intervals.

A comparison of taxa richness between habitats was made using rarefaction curves (Fig. 3). These curves illustrate the sample-based observed (solid line) and extrapolated (dotted line) Mau Tau richness, while 95% confidence intervals are indicated as shaded bands. When comparing richness at the 20 samples mark (total number of samples collected at the RZ habitat), it is clear that RZ presented the greatest richness with 84 genera. This was followed by SRO and OG, which presented 73 and 65 genera, respectively. Nonetheless, it is important to note that these curves did not reach an asymptote (leveling), suggesting that additional sampling might be required to record the complete nematode community. To this end, extrapolation revealed that more than 50 samples per habitat are likely required at which point an estimated 98, 91, and 83 genera would be predicted to be collected at the RZ, SRO, and OG habitats, respectively.

**Figure 4:**
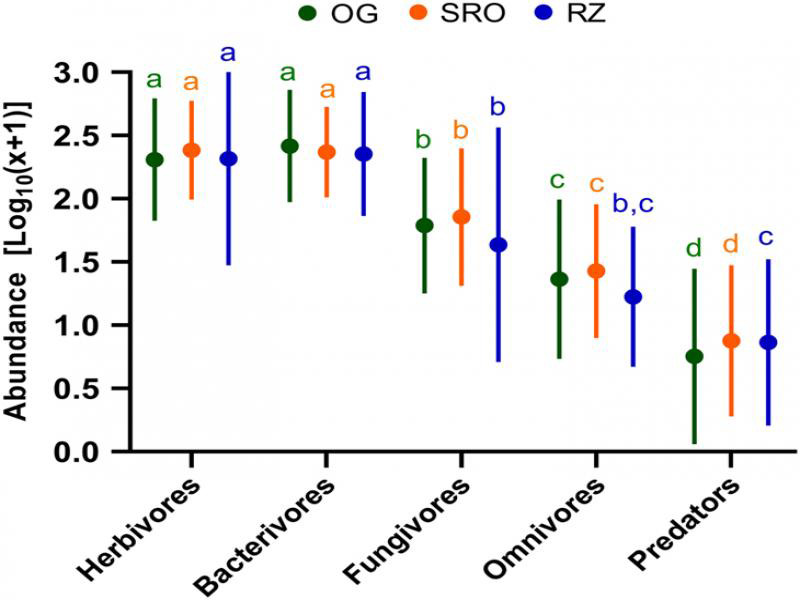
Log_10_(*x* + 1)-transformed nematode abundances (mean, minimum, and maximum) per trophic group at the open grassland (OG), shrubland with rocky outcrops (SRO), and riparian zone (RZ) habitats in Telperion Nature Reserve (South Africa). Bars with common superscript do not differ significantly (*p* > 0.05).

Lastly, the nematode trophic abundances per habitat were considered. Figure 4 shows that herbivores and bacterivores were the most abundant trophic groups, followed by fungivores, omnivores, and predators. The multiple comparisons tests revealed that although no significant differences occurred between herbivores and bacterivores, both these trophic groups differed significantly from fungivores, omnivores, and predators at all three habitats. Also, significant differences between fungivore, omnivore, and predator abundances were recorded at all three habitats with the exception of no significant difference between fungivores and omnivores/predators at the RZ habitat. No significant differences in nematode trophic abundances were recorded between habitats.

### Soil ecosystem condition and food web status

**Figure 5:**
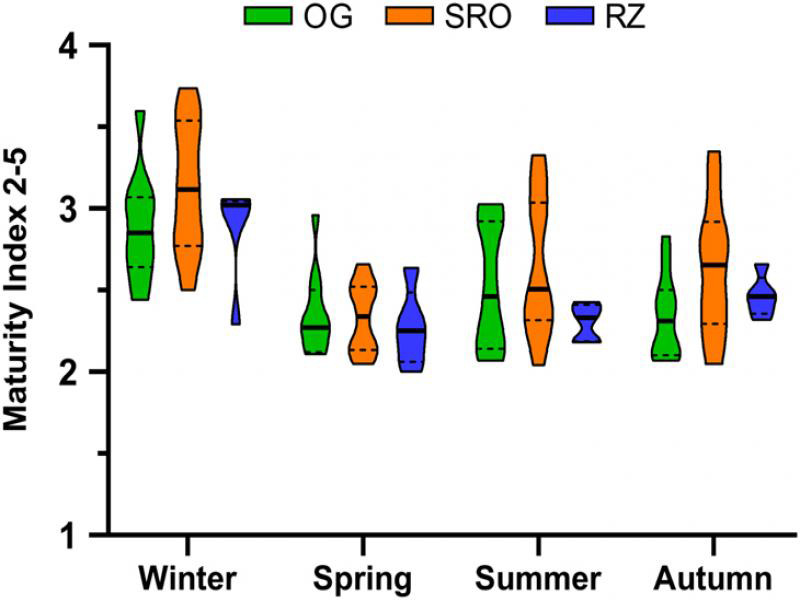
Maturity index 2-5 violin plot of values recorded during the studied seasons at the open grassland (OG), shrubland with rocky outcrops (SRO), and riparian zone (RZ) habitats in Telperion Nature Reserve (South Africa). The bold line within each plot indicates the median value, dashed lines are quartiles, minimum, and maximum are the bounds of the plot and data distribution is shown by plot shape outline.

The maturity index 2-5 was used to investigate the soil ecosystem condition of the studied grassland habitats. A violin plot (Fig. 5) illustrates the median, quartiles, minimum, maximum, and data distribution (shape outline) of this index per habitat per season. Minimum and maximum values ranged from 2 (RZ in Spring) to 3.7 (SRO in Winter), respectively, while the highest and lowest median values for all three habitats were recorded during the Winter and Spring, respectively. Also worth noting is that the SRO habitat had the highest median values during all four seasons. This habitat was thus classified as having the most stable soil ecosystem. The results from the linear mixed models (Table 1) showed that habitat and season had a significant effect on the maturity index 2-5. Yet, pairwise comparisons revealed that these effects were only evident between seasons, which showed significantly higher maturity index 2-5 values in Winter compared to the rest of the seasons. Differences between Spring, Summer, and Autumn were not significant.

**Table 1:** Mixed linear models reveal the effect (singularly and interactively) of the independent variables, season and habitat, on the calculated nematode-specific indices.

Variable	Source	*F* ratio	*p* value	Variable	Source	*F* ratio	*p* value
Maturity index 2–5	Habitat	3.66	*0.04*	Bacterivore footprint	Habitat	0.04	0.96
	Season	14.73	*<0.001*		Season	2.46	0.07
	Habitat × Season	0.42	0.86		Habitat × Season	0.58	0.75
Channel index	Habitat	2.49	0.11	Fungivore footprint	Habitat	0.35	0.71
	Season	11.67	*<0.001*		Season	1.52	0.22
	Habitat × Season	1.38	0.24		Habitat × Season	1.01	0.42
Basal index	Habitat	4.96	*0.01*	Omnivore footprint	Habitat	0.76	0.48
	Season	11.08	*<0.001*		Season	2.54	0.06
	Habitat × Season	0.35	0.91		Habitat × Season	0.29	0.94
Enrichment index	Habitat	8.77	*<0.001*	Predator footprint	Habitat	1.45	0.26
	Season	7.65	*<0.001*		Season	4.53	*<0.01*
	Habitat × Season	1.08	0.38		Habitat × Season	0.71	0.65
Structure index	Habitat	2.00	0.16	Enrichment footprint	Habitat	1.49	0.25
	Season	13.33	*<0.001*		Season	0.29	0.83
	Habitat × Season	0.64	0.70		Habitat × Season	0.84	0.54
Herbivore footprint	Habitat	0.56	0.58	Structure footprint	Habitat	0.11	0.90
	Season	1.60	0.20		Season	4.73	*<0.01*
	Habitat × Season	1.34	0.25		Habitat × Season	0.52	0.79

**Figure 6:**
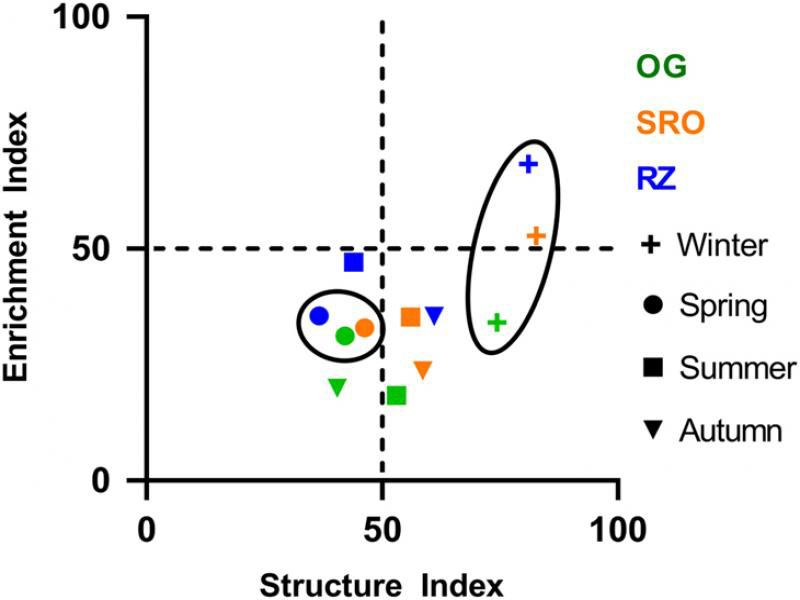
Nematode faunal analysis indicating the food web status during the studied seasons at the open grassland (green), shrubland with rocky outcrops (orange), and riparian zone (blue) habitats in Telperion Nature Reserve (South Africa). Winter and Spring seasonal clusters are circled.

The status of the soil food webs were investigated using the nematode faunal analysis (Fig. 6). This analysis showed that all three habitats had the highest structure (complexity) in the Winter with the SRO and RZ habitats classified as maturing and enriched with balanced (bacterial vs. fungal) decomposition pathways. The OG habitat was classified as mature and fertile characterized by fungal dominated decomposition. In Spring, however, nematode community composition in all three habitats represented degraded and depleted food webs. During Summer and Autumn, the communities represented either degraded and depleted or mature and fertile food webs. The faunal analysis also revealed the clustering of habitats (i.e. low variability between habitats) especially during Winter and Spring. The effect of season and habitat on the two indices used to determine the food web status, namely the structure index and enrichment index (Fig. 6), were further investigated using linear mixed models. This revealed that while both season and habitat had a significant effect on the enrichment index, only season had a significant effect on the structure index. Pairwise comparisons showed, as with the maturity index 2-5, that communities in Winter had significantly higher enrichment and structure compared to the other seasons, while the OG habitat had significantly lower enrichment compared to the SRO and RZ habitats.

**Figure 7:**
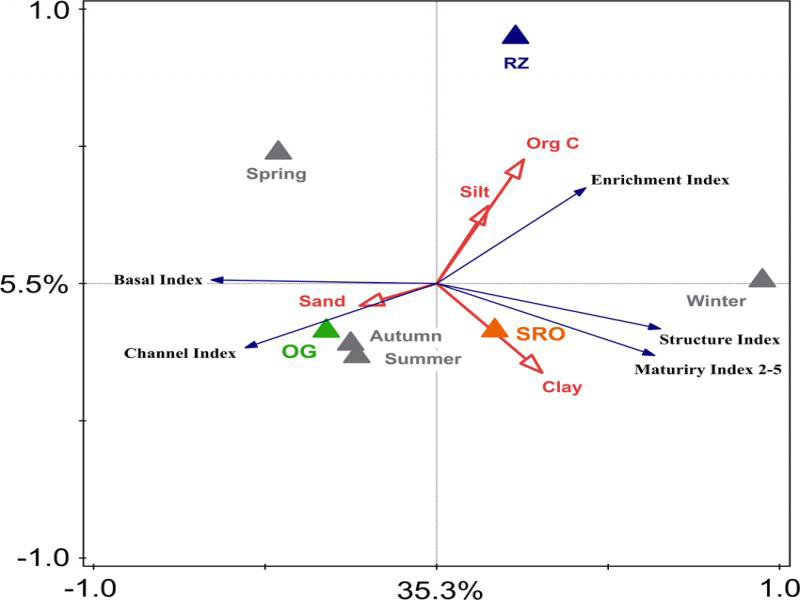
Redundancy analysis of the relationship between the response (nematode-based indices) and explanatory variables (factors: seasons and habitats; numeric variables: soil texture and total organic carbon). Measurements were taken during four consecutive seasons at open grassland (OG), shrubland with rocky outcrops (SRO), and riparian zone (RZ) habitats in Telperion Nature Reserve (South Africa).

Finally, a redundancy analysis illustrated on a biplot (Fig. 7) was used to investigate the relationship between the explanatory and response variables. The explanatory variables accounted for 42% (*p* < 0.001) of the observed variation in the response variables with axes 1 and 2 representing 35.3% (*p* < 0.001) and 5.5% (*p* < 0.001), respectively. This analysis revealed that the three habitats could be differentiated based on the predominant soil fraction, i.e. the OG, RZ, and SRO habitats consisted of more sandy, silty, or clayey soils, respectively. Nonetheless, only clay presented a significant effect of 4.3% on the response variables. In addition, organic carbon levels were positively correlated with the RZ habitat and enrichment index, but had only a minor effect (< 4%) on the response variables. Furthermore, the redundancy analysis biplot showed that a positive correlation existed between the OG habitat and the channel and basal indices. The SRO habitat, in turn, presented a positive correlation to the maturity 2-5 and structure indices. Finally, the Monte-Carlo permutation test revealed that 30% of the variation in response variables can be attributed to seasonality.

### Soil ecosystem functioning

Metabolic footprints (Table 2) were used to assess the soil ecosystem functioning, i.e. the magnitude of functions and services delivered by the soil ecosystems. Generally, relatively high herbivore footprints were recorded, while especially high herbivore footprints were observed in Winter and Spring at the SRO and RZ habitats, respectively. However, even though clear differences were recorded in the herbivore footprint between seasons and habitats, the linear mixed models (Table 1) revealed that none of these differences were significant. Of the remaining footprints, significant seasonal effects were only recorded for the predator and structure footprints. Pairwise comparisons revealed that the predator footprints were significantly higher in the Winter compared to Spring and Summer, while the structure footprints were significantly higher in the Winter compared to Spring and Autumn. No significant habitat effects were recorded for any of the footprints.

**Table 2.** Metabolic footprints calculated from 250 cm^3^ soil samples obtained from riparian zone, shrubland with rocky outcrops and open grassland sites in the Telperion Nature Reserve (Mpumalanga province, South Africa) as part of an ecological study that was conducted over four seasons from 2015 to 2016.

	Open grassland (OG)				Shrubland with rocky outcrops (SRO)				Riparian zone (RZ)			
	Winter	Spring	Summer	Autumn	Winter	Spring	Summer	Autumn	Winter	Spring	Summer	Autumn
Herbivore footprint	120 ± 70	118 ± 119	53 ± 63	168 ± 265	682 ± 1,326	116 ± 111	124 ± 117	116.11 ± 134	97 ± 81	683 ± 1,300	331 ± 326	289 ± 434
Bacterivore footprint	84 ± 49	56 ± 62	50 ± 74	99 ± 87	85 ± 63	91 ± 156	47 ± 52	57.21 ± 30	89 ± 62	66 ± 58	65 ± 67	29 ± 12
Fungivore footprint	14 ± 11	23 ± 26	25 ± 39	9 ± 7	11 ± 6	34 ± 60	23 ± 23	17.56 ± 22	4 ± 4	16 ± 18	49 ± 58	37 ± 55
Omnivore footprint	179 ± 97	103 ± 110	89 ± 94	91 ± 155	123 ± 67	78 ± 69	102 ± 124	58 ± 50	63 ± 34	54 ± 53	89 ± 150	241 ± 477
Predator footprint	12 ± 11	3 ± 4	6 ± 10	11 ± 11	31 ± 26	7 ± 9	7 ± 8	8 ± 8	15 ± 14	4 ± 3	22 ± 42	15 ± 13
Enrichment footprint	16 ± 8	30 ± 40	19 ± 38	21 ± 18	46 ± 65	68 ± 147	24 ± 21	23.46 ± 25	28 ± 26	35 ± 34	51 ± 44	33 ± 55
Structure footprint	217 ± 109	114 ± 112	103 ± 94	107 ± 153	178 ± 62	91 ± 78	122 ± 140	86 ± 62	123 ± 47	66 ± 61	132 ± 140	266 ± 481

**Note:** Mean ± standard deviation values are reported.

## Discussion

### The nematode community structure

Grasslands typically support a high richness of nematode taxa (Song et al., 2017). Yeates and Lee (1997) and Bell et al. (2005) reported 32 and 70 genera, respectively, from tussock grasslands in New Zeeland. Popovici and Ciobanu (2000), in turn, listed between 33 and 67 genera from different grassland regions in Romania, while Čerevková (2006) reported 64 genera in grasslands from the Slovakia Republic. The present study, however, reported a substantially higher number of nematode genera of 93 in total. Rarefaction curves suggest that increased sampling efforts at these sites would result in further taxa being added to that total. At the RZ habitat, for example, extrapolation of the Mau Tao richness indicator suggests that close to a 100 nematode genera could be recovered with increased sampling efforts. The high number of recorded and estimated genera is important as it is commonly accepted that a high richness in nematode taxa are indicative of healthy and functioning soil ecosystems (Ekschmitt et al., 2001; Ferris, 2010; Sánchez-Moreno and Ferris, 2018). A high richness of herbivore taxa in natural environments are often the result of a high diversity in plant species (Hodda et al., 2009; Cortois et al., 2017). According to Hodda et al. (2009), some evidence suggests that a potential reason for this is the lack of competition resulting from root systems creating very defined niches.

Another important consideration when looking at nematode community structure is the abundance distribution between trophic groups (Ferris et al., 2001; Ferris, 2010; Van Den Hoogen et al., 2019). A recent study by Van Den Hoogen et al. (2019) investigated the abundance of nematode trophic groups across all major terrestrial biomes and showed that, on average, bacterivores dominated, followed by herbivores, fungivores, omnivores, and predators. The same trend was observed for temperate grasslands, which is the classification under which the South African grassland biome is listed (Van Den Hoogen et al., 2019). However, even though bacterivores are reportedly the most abundant trophic group on a global and biome scale, studies have reported regional grassland nematode communities with large and even dominating herbivore populations (Porazinska et al., 2003; Ayres et al., 2008). Similarly, during the present study, no significant difference was recorded between the two most abundant groups, namely herbivores and bacterivores, at any of the habitats.

It is worth noting that herbivores of economic importance (e.g. *Meloidogyne*) occurred in relatively high numbers at some sampling sites. Other herbivores (e.g. *Helicotylenchus*, *Rotylenchulus*, and *Scutellonema*) were present in all the habitats during all four seasons. The presence of these herbivore taxa, sometimes in high numbers, poses a threat to crop production in this region. According to various studies conducted in production areas where major grain crops (e.g. maize and soybean) are produced, *Meloidogyne* is generally the most predominant nematode pest. Substantial yield losses in maize (up to 60%) (Riekert and Henshaw, 1998) and soybean (up to 100%) (Smit and De Beer, 1998; Fourie et al., 2013) due to infection by this genus demonstrate its adverse impact on crop production. Moreover, *Helicotylenchus*, *Rotylenchulus*, and *Scutellonema* are also abundant in local crop fields, indicating their potential impact on crop production (Fourie et al., 2001; Bekker et al., 2016; Mbatyoti et al., 2018). Therefore, grasslands may act as reservoirs for herbivores that pose a threat to crop production should such areas be converted into agricultural fields.

### Seasonal and habitat induced changes in grassland ecosystems

Seasonality had a large and significant effect on the condition, food web status and functioning of the studied grassland soil ecosystems. This effect was most evident progressing from Winter to Spring. During Winter the habitats were enriched with stable ecosystems and structured food webs, while depletion with reduced stability and structure were evident in Spring. Furthermore, during the Winter increased ecosystem functioning were evident with significantly higher predator and structure footprints. This even though low temperatures and reduced precipitation (as recorded during the 90 days period prior to Winter sampling) typically exert a negative influence on nematode communities (Verschoor et al., 2001; Song et al., 2017; Andriuzzi et al., 2020). According to Song et al. (2017), the optimal temperature for the survival and reproduction of soil nematodes ranges from 20 to 25°C, while extremes below 5 and above 30°C significantly inhibit development. Similarly, water availability influences primary production and thus energy flow into the soil food web, while also regulating organic carbon decomposition (Andriuzzi et al., 2020). It should be noted that the especially high herbivore footprints can be attributed to the large number of *Meloidogyne* that were present in some of the samples. Metabolic footprints are calculated using the biomass of adult females (Ferris, 2010), which can substantially increase footprint values if large numbers of, for example, some sedentary endoparasitic nematodes are recorded.

In contrast to seasonal effects, habitats and the measured soil properties had minimal influence on the studied soil ecosystems. This despite habitat type, soil texture and organic carbon levels being known to substantially influence soil faunal communities (Du Preez et al., 2018; Sánchez-Moreno and Ferris, 2018; Sprunger et al., 2019). Nonetheless, the positive correlation (as evidenced by the redundancy analysis) between organic carbon and the enrichment index is in line with the general understanding that higher organic carbon levels support opportunistic bacterial-feeding nematodes (Sánchez-Moreno and Ferris, 2018). Increased levels of soil organic carbon at the RZ habitat is therefore likely the reason for this habitat also having the highest nematode community enrichment levels during all four seasons in the soil food web analysis. Conversely, the basal and channel indices were positively correlated with the OG habitat, indicating that soil ecosystems associated with this habitat were likely more degraded, but with greater fungal decomposition (Ferris et al., 2001; Ferris, 2010; Sánchez-Moreno and Ferris, 2018).

Interestingly, the abundance of nematode taxa (Supplementary material: Tables S2–S6) made it clear that the dramatic shift in nematode community in Winter can largely be attributed to fluctuations in the populations of only a few predacious nematodes (namely *Aporcelaimellus*, *Iotonchus*, and *Mononchus*), as well as one omnivorous nematode group, the Dorylaimidae. These taxa are assigned a colonizer-persister (c-p) value of 4 (Dorylaimidae and *Iotonchus*) and 5 (*Aporcelaimellus* and *Mononchus*) (Sieriebriennikov et al., 2014), which means that they are regarded as persisters (or k-strategists). Their presence in large numbers therefore infers greater ecosystem stability and food web structure (Ferris et al., 2001; Sánchez-Moreno and Ferris, 2018), as was recorded during Winter at the SRO and OG habitats. The reason for the increased number of members of these nematode groups recorded during Winter is not explicitly clear, however, findings from previous studies provide some potential answers. According to De Ley (1992), desert and dune sands often contain a remarkably high proportion of dorylaims, while Arpin (1969) repeatedly found dorylaims (predators and omnivores) in desiccation experiments. De Ley and Mundo-Ocampo (2004), in turn, stated that the ecological sensitivity of these nematodes may have been overestimated and that certain species may be capable of surviving desiccation and freezing. Omnivorous nematodes typically also have versatile feeding habits and can probably interact at various levels of the soil food web (Hanel, 2003). It is therefore possible that dorylaims in the studied Rand Highveld Grasslands of South Africa are not adversely affected by the conditions present in Winter and may even increase in numbers following reduced competition for resources from other faunal groups. The possibility that certain nematode families and genera are favored by Winter conditions (low temperature and rainfall), infers that nematode community responses to altered conditions are taxa dependent (see also Papatheodorou et al., 2004).

## Conclusion

The grassland habitats of Telperion Nature Reserve host a diversity of nematode taxa, many of which are likely still to be observed or described. Although the studied nematode communities were dominated by herbivores and bacterivores, it was the less abundant nematode groups, i.e. omnivores and predators, which had a significant influence on the condition, food web status, and functioning of the soil ecosystems. Furthermore, evidence suggests that only a few taxa are largely responsible for seasonal shifts in measurable soil ecosystem parameters.

This study also recorded a high abundance in nematodes of economic importance, which has important implications for crop production in this grassland biome.
